# Supplementary material for: Uncovering miRNA-mRNA Regulatory Modules in Developing Xylem of Pinus massoniana via Small RNA and Degradome Sequencing
Source: Int J Mol Sci. 2021 Sep 21;22(18):10154. doi: 10.3390/ijms221810154 (PMC8472836; doi:10.3390/ijms221810154)
Supplement: Supplementary file 1 [file ijms-22-10154-s001.zip › ijms-1365557-supplementary/Additional file S2.zip!/Additional file 2/novel_78_Cluster-18410.26251_232_TPlot.pdf]

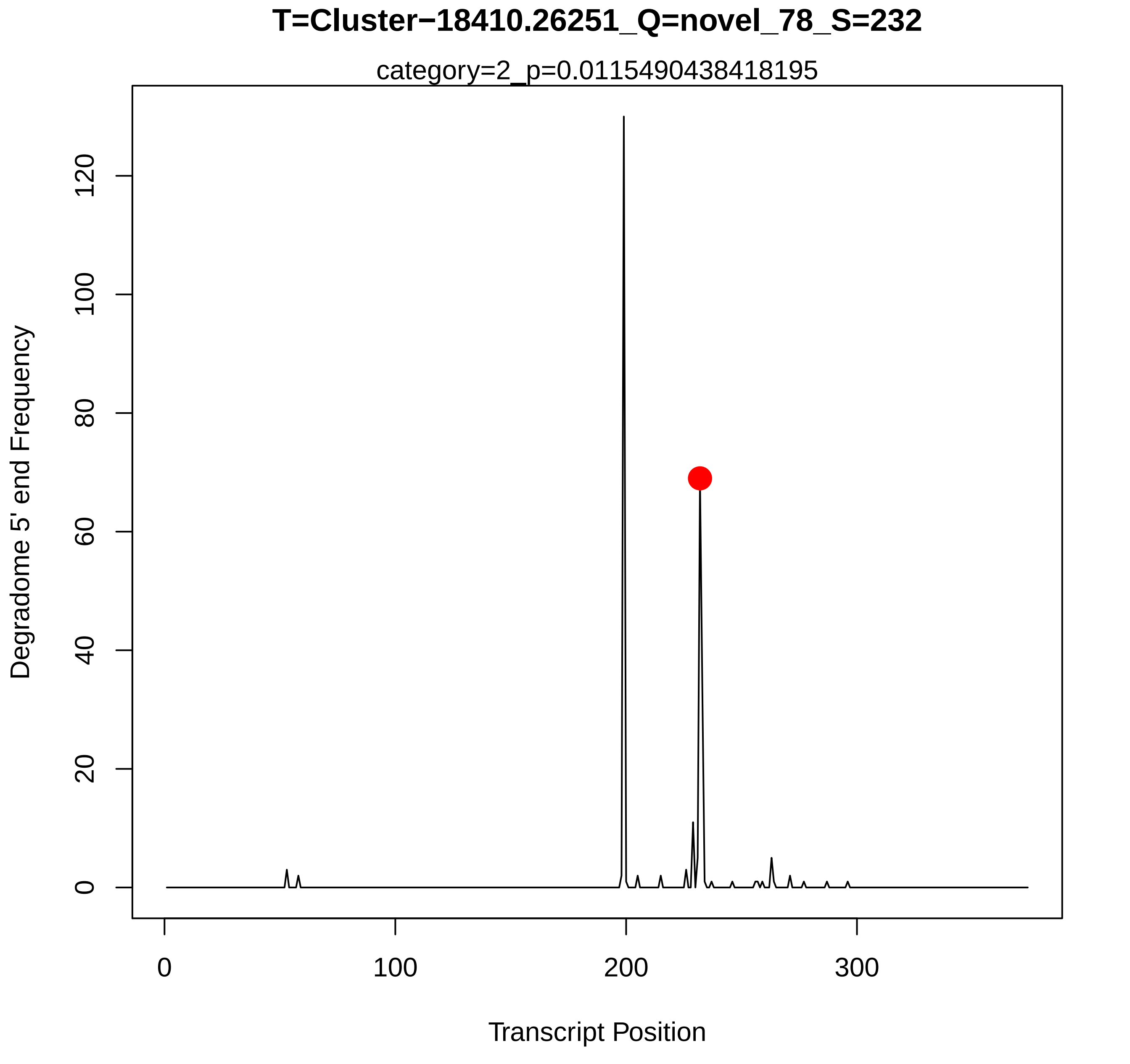
T=Cluster−18410.26251_Q=novel_78_S=232
category=2_p=0.0115490438418195
Degradome 5' end Frequency
0
20
40
60
80
100
120
0
100
200
300
Transcript Position
